# Impressive reduction of brain metastasis radionecrosis after cabozantinib therapy in metastatic renal carcinoma: A case report and review of the literature

**DOI:** 10.3389/fonc.2023.1136300

**Published:** 2023-03-07

**Authors:** Jacopo Lolli, Francesca Tessari, Franco Berti, Marco Fusella, Davide Fiorentin, Davide Bimbatti, Umberto Basso, Fabio Busato

**Affiliations:** ^1^ Radiotherapy Unit, Veneto Institute of Oncology IOV – IRCCS, Padua, Italy; ^2^ Department of Radiation Oncology, Abano Terme Hospital, Padua, Italy; ^3^ Medical Oncology 1, Veneto Institute of Oncology IOV-IRCSS, Padua, Italy

**Keywords:** cabozantinib, radionecrosis (RN), stereotacic radiation, immunotherapy, radiosurgery

## Abstract

**Introduction:**

Radionecrosis is a consequence of SRS (stereotactic radiosurgery) for brain metastases in 34% of cases, and if symptomatic (8%–16%), it requires therapy with corticosteroids and bevacizumab and, less frequently, surgery. Oncological indications are increasing and appropriate stereotactic adapted LINACs (linear accelerators) are becoming more widely available worldwide. Efforts are being made to treat brain radionecrosis in order to relieve symptoms and spare the use of active therapies.

**Case presentation:**

Herein, we describe a 65-year-old female patient presenting with brain radionecrosis 6 months after stereotactic radiotherapy for two brain metastatic lesions. Being symptomatic with headache and slow cognitive-motor function, the patient received corticosteroids. Because of later lung progression, the patient took cabozantinib. An impressive reduction of the two brain radionecrosis areas was seen at the brain MRI 2 months after the initiation of the angiogenic drug.

**Discussion:**

The high incidence of radionecrosis (2/2 treated lesions) can be interpreted by the combination of SRS and previous ipilimumab that is associated with increased risk of radionecrosis. The molecular mechanisms of brain radionecrosis, and its exact duration in time, are poorly understood. We hypothesize that the antiangiogenic effect of cabozantinib may have had a strong effect in reducing brain radionecrosis areas.

**Conclusion:**

In this clinical case, cabozantinib is associated with a fast and significant volume reduction of brain radionecrosis appearing after SRS and concomitant immunotherapy. This drug seems to show, like bevacizumab, clinical implications not only for its efficacy in systemic disease control but also in reducing brain radionecrosis. More research is needed to evaluate all molecular mechanisms of brain radionecrosis and their interaction with systemic therapies like third-generation TKIs.

## Introduction

Stereotactic radiotherapy is a type of external radiation therapy that uses special equipment to position the patient and precisely give a high radiation dose to a tumor. It generally consists of less than five fractions, and it is also called hypofractionated stereotactic radiotherapy. When the radiation is delivered in one single fraction, it is called stereotactic radiosurgery (SRS).

Thanks to clinical trials and retrospective data (1, 2), stereotactic radiation therapy has almost replaced whole brain irradiation in case of multiple brain metastases. Whole brain remains a therapeutic option, eventually associated with hippocampal sparing (3), in case of multiple brain lesions (>10), uncontrolled extracranial disease, and high cumulative central volume (>15 ml) (2).

Stereotactic radiotherapy is associated with 85%–95% local control at 1 year for lesions <2 cm (4, 5) and very few adverse events (6). One of the major adverse events is brain radionecrosis.

Radionecrosis is a focal structural anomaly that forms following cranial irradiation of cerebral neoplasms, and it is observed in approximately 5%–26% of patients at 1 year and up to 25.5%–34% at 2 years of follow-up (7–11). It can be asymptomatic in approximately 14%–84% of cases and symptomatic in approximately 8.4%–16.4% of cases (12–14).

The time frame of presentation is variable and typically ranges from 3 months to 10 years post-radiotherapy, but 80% of cases occur within 3 years after the completion of radiotherapy (15).

Radionecrosis is treated with the lowest dose of corticosteroids and, if the patient becomes refractory or intolerant to corticosteroids, surgery and bevacizumab are discussed at multidisciplinary boards (16, 17).

## Case presentation

A 65-year-old woman was treated with radical left nephrectomy and lymph node dissection in 2018. The pathological diagnosis was clear cell renal carcinoma, grade 2, pT3a-pN0. The full-body CT showed no metastatic lesions at baseline (cM0).

After 3 months, the full-body CT showed progression in multiple lung and abdominal lymph nodes; she therefore started first-line systemic therapy with nivolumab and ipilimumab for 3 months, followed by nivolumab as maintenance therapy. Because of the progression of lung, wedge resection was performed for two pulmonary lesions in 2020 after multidisciplinary board decision.

**Figure 1:**
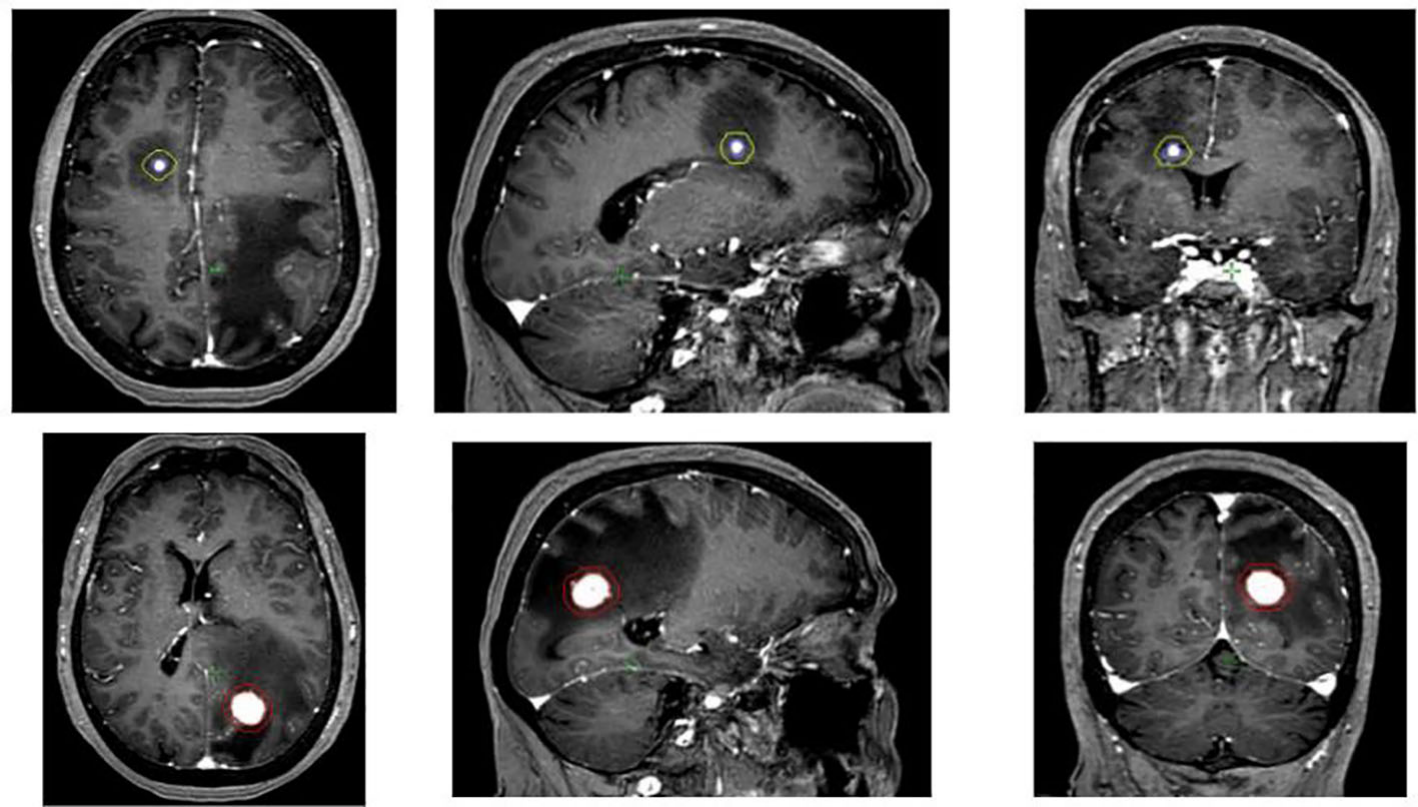
Brain MRI showing the two brain metastases, the right frontal (3 mm) and the left occipital one (18 mm). GTV was the T1-gadolinium enhanced lesion while PTV was defined as GTV + 3 mm.

In October 2021, brain MRI revealed two brain metastasis, one was in the right frontal lobe (3 mm) and the other in the left occipital lobe (18 mm), both associated with extensive brain edema (**Figure 1**). The patient was admitted to the hospital for headache and asthenia, limiting deambulation. Stereotactic radiotherapy was performed on both lesions. The frontal lesion was treated with 20 Gy/1 fraction on PTV (planning tumor volume) and 25 Gy on GTV (gross tumor volume), with respect to V12 Gy of brain including target < 5 cm^3^ and V14 Gy < 5 cm^3^; the occipital lesion was treated with 27 Gy/3 fractions on PTV and 33 Gy on GTV, with respect to V20 Gy of brain including target < 20 cm^3^ and V14 Gy < 20 cm^3^ (18); nivolumab was reestablished soon after the tapering of corticosteroids. A 3-mm GTV–PTV margin was considered appropriate by physicians and physicists as voluminous edema and high-dose corticosteroids could dislocate the GTV, and because of setup accuracy when performing SRS with adapted LINAC. The specific prescription to GTV/PTV with respect to radiation necrosis constraints, as described, was done according to the institute’s clinical practice.

The 1-month (November 2021) and 3-month brain MRI (January 2022) showed reduction of the occipital lesion but increased contrast enhancement area of the frontal lesion associated with edema in T2-FLAIR (Fluid Attenuated Inversion Recovery) sequence: being symptomatic with headache and cognitive-motor slowing, mannitol and high-dose corticosteroids were administered in Day Hospital.

As for extracranial single-site disease progression, stereotactic radiotherapy was delivered to a pancreatic lesion (35 Gy/5 fractions on PTV) in February 2022, in addition to nivolumab.

In April 2022, the brain MRI revealed partial response of the occipital metastasis and suspected frontal radionecrosis (mismatch T2/T1, necrotic and bubble-like lesion in T1-contrast sequence) and no new lesions.

In July 2022, the brain MRI showed increased contrast enhancement of the central lesions, both suspected for brain radionecrosis. The case was discussed several times at the neurooncological multidisciplinary team: surgery was not suggested as it was neither considered safe (deep parenchymal lesions) nor advisable considering the clinical partial benefit obtained by corticosteroids. Therefore, pathological confirmation of the diagnosis was not possible to achieve. A radiological diagnosis of brain radiation necrosis was maintained considering the radiological and clinical evolution of the lesion during the 8-month follow-up period. A neuro-radiologist was always present at multidisciplinary team meetings for a qualified review of images (DWI, T2, T1 with contrast, FLAIR, STIR, ADC, and perfusion with CBV sequences).

In September 2022, abdomen and thoracic CT scan pointed out a renal and pulmonary disease progression and the patient started second-line therapy with cabozantinib.

**Figure 2:**
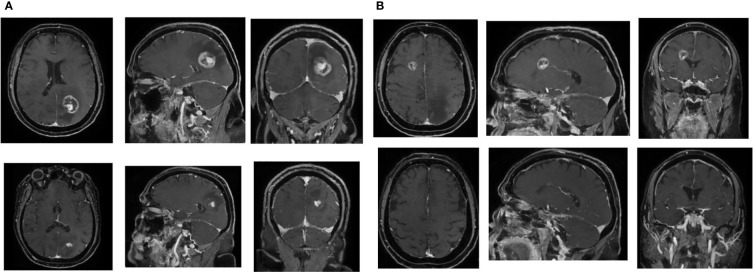
Impressive volume reduction of two brain radionecrosis areas. **(A)** Left occipital lesion. Above, MRI before cabozantinib (9 months after SRS). Below, MRI after cabozantinib (13 months after SRS). **(B)** Right frontal lesion: Above, MRI before cabozantinib (9 months after SRS). Below, MRI after cabozantinib (13 months after SRS).

After only 2 months from second-line systemic therapy, the brain MRI showed excellent reduction of the two areas of brain radionecrosis with a surprising shrinkage of the associated edema (**Figure 2**). The patient is still alive and is being treated with cabozantinib when this article is sent to publication.

The patient has given written consent to the publication of images and personal clinical data for this case report description.

## Discussion

The incidence of brain radionecrosis after stereotactic radiotherapy for brain metastases is up to 34% (19). In our case, the incidence was higher as both treated lesions presented with signs of radionecrosis at control brain MRI. The increased incidence we observe can be interpreted by data available in the medical literature.

Firstly, we reviewed the dosimetric constraints and verified that they had been respected (18). Although a clinical trial with 49 patients has demonstrated that 1-mm margin PTV is associated with a lower risk of radionecrosis when compared to 3-mm margin PTV (20), and the same authors indicate that a 1-mm GTV–PTV margin would be ideal, but possible uncertainties such as patient setup and accuracy should be considered when choosing the margin. The reasons why we considered a 3-mm GTV–PTV margin appropriate are both clinical and dosimetric. From a clinical point of view, as shown in MRI figures, both lesions were surrounded by voluminous edema that warranted high-dose corticosteroids that could shift the target position. From a physicist point of view, radiosurgery with multi-source gamma-ray platforms requires no expansions but radiosurgery delivered by stereotactic adapted LINACs, as in our case, requires expansions to take into consideration possible uncertainties in patient setup, beam alignment, organ motion, organ deformation, and planning accuracy (fusion of images, contrast medium acquisition time, etc.). Despite the different margins recommended, there are no differences in the rate of symptomatic radionecrosis between adapted LINACs and multi-source gamma-ray platforms (21).

Secondly, we reviewed data about SRS and immunotherapy. In fact, some authors have hypothesized that concomitant immunotherapy, by promoting T-cell activation and anti-tumor response, may trigger radionecrosis (22, 23). Association between radionecrosis and previous ipilimumab has already been demonstrated (31% versus 13%) (24, 25). SRS in combination with ipilimumab was also observed to cause a temporary increase in lesion diameter to >150% (26).

Few data are available about the association between dual immunotherapy and radiation necrosis: though Johnson et al. (27) demonstrated that the dual immunotherapy (ipilimumab + nivolumab) is not associated with higher incidence of radiation necrosis, data in the literature often make no specification about radionecrosis diagnostic criteria, grading, and radiation dose prescription.

An impressive volume reduction was observed soon after the initiation of cabozantinib, an antiangiogenic drug. Antiangiogenic drugs have been used against RCC (renal cell carcinoma) for more than a decade. They are classified as first-generation TKIs (tyrosine kinase inhibitors), such as sunitinib, sorafenib, and pazopanib, and second-generation TKIs, such as axitinib and tivozanib, and, later, cabozantinib and envatinib (28).

**Figure 3:**
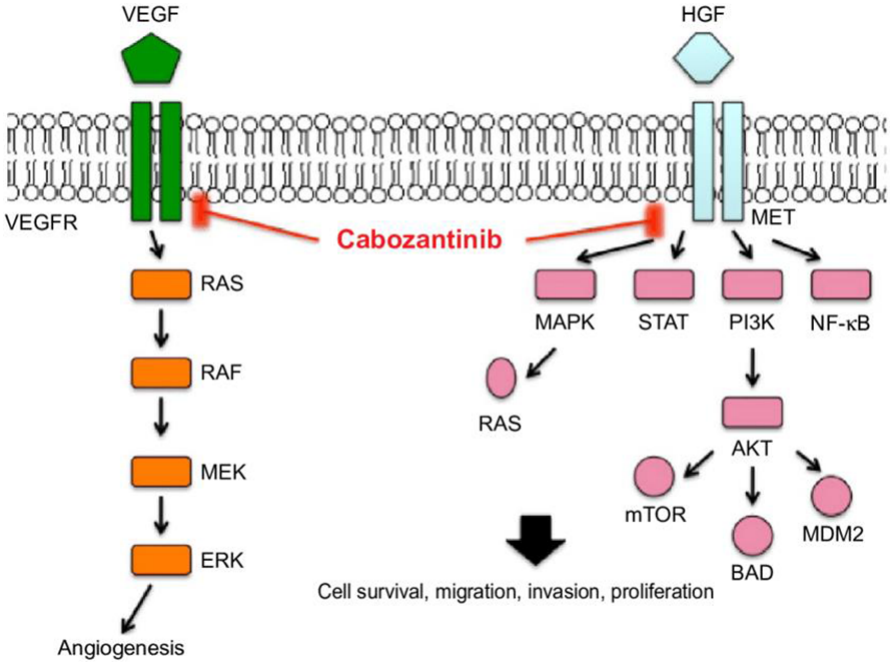
Molecular pathways of cabozantinib [image from Yu et al. (31)].

Cabozantinib is an oral TKI targeting several receptors involved in angiogenesis pathways such as VEGFR2, c-MET, RET, c-Kit, AXL, TIE2, ROS1, TYRO3, MERTK, TRKB, and FLT3 receptors. All of these targets and pathways are implicated in cancer development and progression (29). Inhibition of the VEGFR blocks angiogenesis, cell tubule formation, cellular migration and invasion, and cell proliferation, and induces apoptosis (30). Cabozantinib is a RET inhibitor: the RET receptor is implicated in the inflammation process, with its activation resulting in an increase of cytokines in the tumor microenvironment, leading to the recruitment of suppressive immune cells and, thus, allowing tumor growth and invasion (**Figure 3**).

After first-line nivolumab–ipilimumab therapy, the median time to failure for cabozantinib is 6.90 months (32) with a median OS of 21.44 months and 37.9% of ORR (33). More data of cabozantinib as a second-line therapy will be revealed by the ongoing trial CaboPoint (34). While the association between cabozantinib and SRS has proved to be safe (35), limited data about the effect of cabozantinib started on active brain radionecrosis are available.

**Figure 4:**
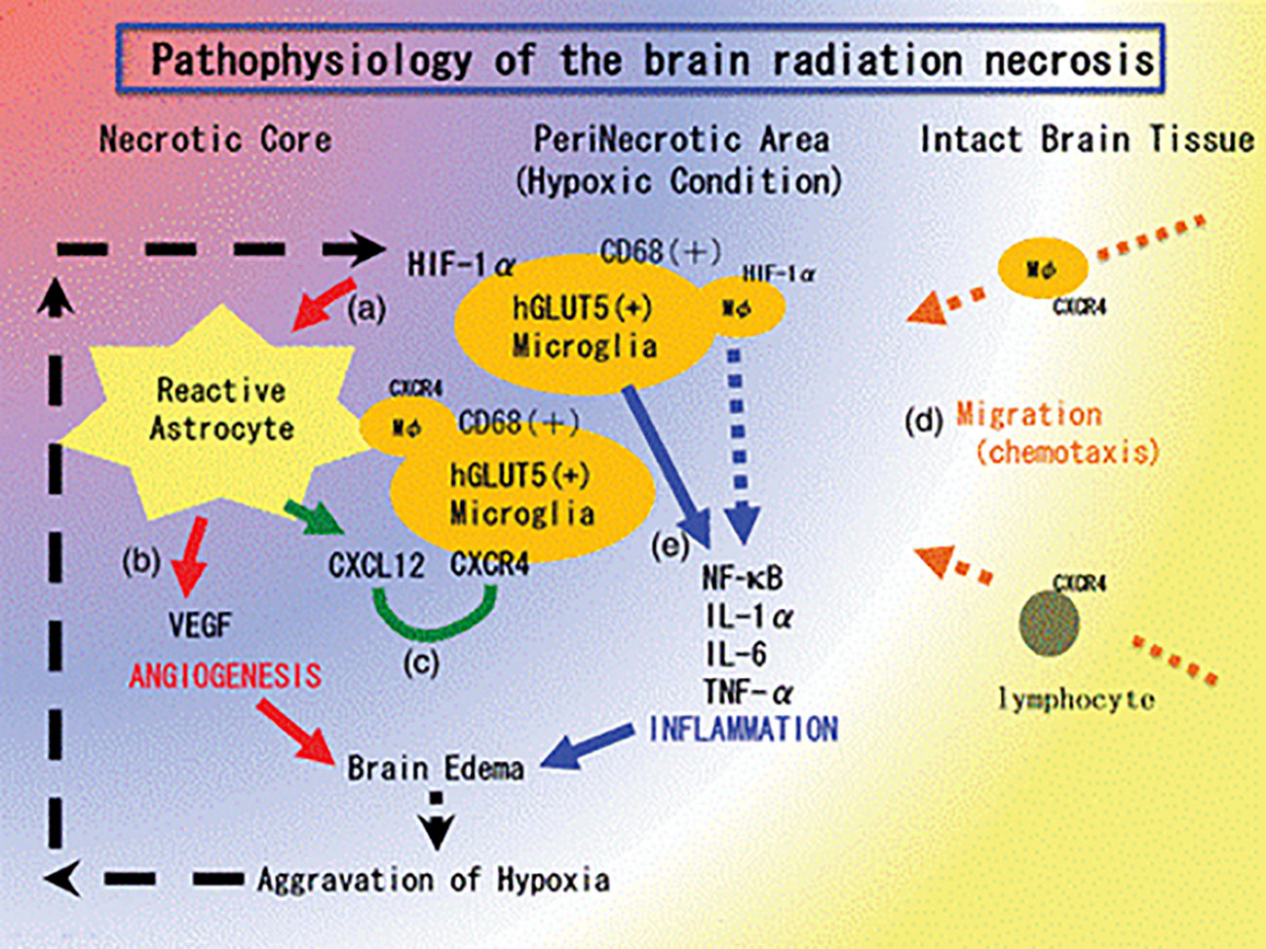
The pathophysiology of brain radiation necrosis from Yoritsune et al. (36).

Currently, molecular mechanisms of radionecrosis are not fully understood. The principal hypothesis holds that stereotactic radiotherapy is responsible for direct damage to the blood vessels around the irradiated area as demonstrated in human specimens (36). This damage causes hypoxia around the irradiated area and, thus, upregulation of the hypoxia-inducible factor-1 alpha (HIF-1-alpha) in glial cells (37). HIF-1-alpha augments VEGF, which is responsible for the neo-angiogenesis and subsequent brain edema. HIF-1-alpha also augments inflammatory cytokines (IL-1, IL-6, and TNF alpha) that seem to aggravate the perilesional edema (**Figure 4**) (38).

The inhibition triggered by cabozantinib is fundamentally arresting the neoangiogenesis cascade and the fibroblast activation that is finally responsible for tissue fibrosis and brain radionecrosis. In addition, cabozantinib is a selective inhibitor of C-MET that is overexpressed in clear cell carcinoma tumors, and this aspect shows that this drug could have a direct tumoricidal effect, particularly when radionecrosis seems to be accompanied by residual disease (39). These effects are possible thanks to the drug pharmacokinetics as cabozantinib crosses the blood–brain barrier: some rodent models revealed that cabozantinib concentration in brain reaches 20% of the peak plasma level (40, 41).

This case report has some limitations. First of all, in the absence of pathological confirmation, we cannot exclude the persistence of disease in the differential diagnosis with radiation-induced inflammation. After neuro-oncological multidisciplinary discussion, surgery was neither considered safe (deep parenchymal lesions) nor advisable, considering the clinical partial benefit obtained by corticosteroids. The radiological presentation, the time duration (8 months), and the partial response after corticosteroids seem to suggest radionecrosis, although this cannot be stated with certainty.

A second limitation might be considered the 3-mm GTV–PTV expansion instead of a smaller one: voluminous edema, setup accuracy, and a stereotactic-adapted LINAC were the reasons why a 3-mm GTV–PTV margin was considered appropriate, simultaneously respecting constraints as described (brain V12 Gy and V14 Gy) (18).

## Conclusion

In this clinical case, cabozantinib is associated with a fast and significant volume reduction of brain radionecrosis that appeared after radiosurgery and concomitant immunotherapy. This drug seems to show, like bevacizumab, clinical implications not only for its efficacy in systemic disease control but also in reducing brain radionecrosis. More research is needed to evaluate all molecular mechanisms of brain radionecrosis and their interaction with systemic therapies like third-generation TKIs.

## Data availability statement

The raw data supporting the conclusions of this article will be made available by the authors, without undue reservation.

## Ethics statement

Written informed consent was obtained from the individual(s) for the publication of any potentially identifiable images or data included in this article.

## Author contributions

Authors JL, FT and FaB contributed equally. All authors contributed to the article and approved the submitted version.
