# Corrigendum: Impressive reduction of brain metastasis radionecrosis after cabozantinib therapy in metastatic renal carcinoma: a case report and review of the literature

**DOI:** 10.3389/fonc.2023.1278074

**Published:** 2023-09-13

**Authors:** Jacopo Lolli, Francesca Tessari, Franco Berti, Marco Fusella, Davide Fiorentin, Davide Bimbatti, Umberto Basso, Fabio Busato

**Affiliations:** ^1^Radiotherapy Unit, Veneto Institute of Oncology IOV – IRCCS, Padua, Italy; ^2^Department of Radiation Oncology, Abano Terme Hospital, Padua, Italy; ^3^Medical Oncology 1, Veneto Institute of Oncology IOV-IRCSS, Padua, Italy

**Keywords:** cabozantinib, radionecrosis (RN), stereotacic radiation, immunotherapy, radiosurgery

In the published article, there was an error in the Funding statement as it was not specified that this research was funded by the Italian Ministry of Health Ricerca Corrente. The correct Funding statement appears below.

The authors apologize for this error and state that this does not change the scientific conclusions of the article in any way.

